# Integrating TMAO into the pathogenesis of obesity and type 2 diabetes: a mini review

**DOI:** 10.3389/fcdhc.2026.1765794

**Published:** 2026-01-28

**Authors:** Dana Stoian, Denisa Pescari, Andreea Bena, Corina Paul, Simina Mihuta

**Affiliations:** 1 Discipline of Endocrinology, Second Department of Internal Medicine, Victor Babeş University of Medicine and Pharmacy, Timişoara, Romania; 2 Department of Advanced Ultrasound, DRD Medical Center, Timişoara, Romania; 3 Center for Molecular Research in Nephrology and Vascular Disease, “Victor Babeş” University of Medicine and Pharmacy, Timişoara, Romania; 4 Department of Doctoral Studies, “Victor Babeş” University of Medicine and Pharmacy, Timişoara, Romania; 5 Department of Pediatrics, “Victor Babeş” University of Medicine and Pharmacy, Timişoara, Romania

**Keywords:** cardiometabolic disease, diabetes, obesity, TMAO, type 2 diabetes

## Abstract

Increasing circulating levels of trimethylamine N-oxide (TMAO), a metabolite originating from both dietary sources and microbial metabolism in the gut, have drawn significant attention for their possible contribution to cardiometabolic disorders, such as type 2 diabetes and carotid intima–media thickening in individuals with excess weight. Yet, evidence from longitudinal and retrospective investigations remains inconsistent, with studies often reaching divergent conclusions. Higher circulating TMAO levels have been observed in people with overweight, obesity, and type 2 diabetes who subsequently develop cardiovascular complications, as well as in diabetic individuals who experience renal impairment. Still, the strength and consistency of these links vary considerably between cohorts and are shaped by how thoroughly studies control for underlying confounders. Given that insulin modulates FMO3 activity within hepatocytes, circulating TMAO concentrations might reflect underlying hepatic insulin resistance, offering a possible mechanistic link to its reported associations with diabetes and cardiovascular risk. This mini review summarizes current evidence on TMAO in obesity and type 2 diabetes, highlighting the novel comparison between pediatric and adult findings. This approach may help clarify how TMAO-related mechanisms evolve across the lifespan and inform future risk assessment and prevention strategies.

## 1 Introduction

Trimethylamine N-oxide (TMAO) has emerged as a key metabolite at the intersection of habitual diet, host metabolism, and gut microbiome activity, gaining increasing attention as a potential contributor to cardiometabolic disease risk (1, 2). Dietary nutrients commonly consumed in Western-type dietary patterns, particularly L-carnitine and choline, undergo microbial conversion in the intestine to trimethylamine (TMA) (3, 4), which is subsequently oxidized in the liver by flavin-containing monooxygenase 3 (FMO3) to form TMAO (5, 6). This metabolic pathway represents a fundamental mechanistic link between diet-driven microbial metabolism and systemic metabolic regulation, with growing relevance for nutrition-based therapeutic strategies (1, 7).

Accumulating evidence has linked elevated circulating TMAO concentrations to major metabolic disorders, including obesity and type 2 diabetes mellitus (8, 9). Proposed mechanisms include disturbances in lipid metabolism, impairment of insulin signaling, and promotion of chronic low-grade inflammation (10, 11). Several adult studies have reported positive associations between TMAO levels and body mass index, visceral adiposity, and markers of arterial stiffness in individuals with excess weight (12, 13). Together, these observations have positioned TMAO as both a potential biomarker of metabolic dysfunction and a candidate mediator involved in the pathophysiology of cardiometabolic disease, although the relative contribution of these roles remains under debate.

Importantly, the current body of evidence is heavily weighted toward adult populations, while data in children and adolescents remain limited and fragmented (14). This imbalance is particularly relevant in the context of obesity, which affects both adults and younger age groups and continues to rise globally as a major public health concern (15). Early metabolic alterations, including insulin resistance, endothelial dysfunction, and subclinical vascular changes, have already been documented in pediatric populations with obesity (12). However, whether TMAO-related pathways are similarly activated in children, or whether they evolve progressively across the life course, remains insufficiently explored.

In this context, the direct comparison of pediatric and adult data represents a critical and underdeveloped area of investigation. Evaluating TMAO concentrations and associated metabolic features across age groups may offer unique insights into the temporal dynamics of diet–microbiota–host interactions, helping to clarify whether TMAO reflects early metabolic vulnerability or predominantly mirrors cumulative exposure to cardiometabolic risk factors over time (16). Such a life-course perspective is essential for understanding the potential role of TMAO in the early origins of metabolic disease and its utility in risk stratification and prevention strategies.

Accordingly, this mini review synthesizes current evidence on the involvement of TMAO in obesity and type 2 diabetes, with a deliberate and novel emphasis on contrasting findings from pediatric and adult studies. By integrating data across age groups, this review aims to highlight both shared and distinct patterns in TMAO-related metabolic alterations and to identify gaps that warrant further longitudinal and mechanistic research.

From a mechanistic standpoint, TMAO production occurs along the diet–microbiota–liver axis. The process is initiated by ingestion of dietary precursors containing trimethylamine moieties, including choline, phosphatidylcholine, betaine, and L-carnitine, derived primarily from eggs, dairy products, red meat, and certain fish and seafood (3, 17, 18). Within the intestinal lumen, anaerobic bacteria convert these compounds into TMA through well-characterized enzymatic pathways (4, 19). Choline metabolism is mediated by the choline TMA-lyase CutC, activated by the radical-SAM enzyme CutD, while L-carnitine conversion involves the Rieske-type carnitine monooxygenase/reductase system CntA/CntB (20, 21). Additional pathways, including γ-butyrobetaine utilization and homologous gene clusters, contribute variably depending on the host’s microbial composition (19, 20). After intestinal absorption, TMA is transported via the portal circulation and oxidized predominantly by hepatic FMO3 to form TMAO (4, 22). FMO3 expression is influenced by genetic factors, hormonal regulation, and nutritional status, contributing to inter-individual variability in circulating TMAO levels observed in both adults and children (23, 24). Notably, fish consumption provides not only precursors but also preformed TMAO, resulting in marked postprandial increases independent of microbial metabolism (25, 26). Renal excretion represents the primary elimination pathway, making kidney function a major determinant of systemic TMAO exposure (27, 28). Collectively, these mechanisms underscore the complex interplay between diet, gut microbiota, hepatic metabolism, and renal clearance in shaping the TMAO phenotype across different stages of life.

## 2 Assessment of TMAO: serum versus urinary measurements

Quantification is performed predominantly by isotope-dilution liquid chromatography–tandem mass spectrometry, employing a deuterated internal standard (29, 30). This approach ensures accuracy and correction for matrix effects; separation is frequently achieved on hydrophilic-interaction liquid-chromatography columns for highly polar analytes (31). In addition, analytical validations have demonstrated low limits of detection, excellent linearity, and adequate analyte stability at −80 °C and after multiple freeze–thaw cycles (inter-cycle coefficient of variation < 10%) (29, 31, 32).

Plasma TMAO is useful for etiological research and for cardiometabolic risk prediction (33). Its concentrations are influenced to some extent by recent dietary intake, most notably elevated after consumption of fish or seafood, by fasting status, and, most importantly, by renal function, a major driver of inter-individual variability (26, 34). Accordingly, blood sampling is recommended in the fasting state after at least eight hours without food, and results should be interpreted with adjustment for the estimated glomerular filtration rate (35, 36). The latter correlates inversely with TMAO (higher concentrations with reduced renal function), necessitating rigorous control for confounding by renal impairment when interpreting results (37). Beyond renal function, circulating TMAO levels are also modulated by habitual dietary patterns, gut microbiota composition, and host-related factors such as age and sex, which should be considered when comparing results across populations or study designs.

Urinary TMAO quantifies excretion and recent exposure, functioning as a sensitive biomarker of short-term dietary intake; concentrations rise consistently after fish consumption (25, 38). Twenty-four-hour urine collections provide more accurate estimates of total body load than spot samples. In practice, creatinine-normalized spot urine is more feasible but more susceptible to variation in hydration status; therefore, for diet–biomarker studies the twenty-four-hour collection remains the reference standard, whereas spot sampling is useful for population surveillance or for repeated intervention monitoring (39–41). Interpretation of urinary TMAO similarly requires consideration of dietary intake, renal handling, and intra-individual variability related to hydration and collection protocol.

Nevertheless, a range of preanalytical and standardization factors, such as the interval to processing, the temperature at which the specimen is stored, and the number of freeze–thaw cycles, can influence the measurement outcome. Under standard laboratory conditions, both plasma and urinary TMAO appear stable. However, meaningful comparison across studies requires harmonized procedures, including an explicit choice of specimen type, appropriate storage conditions, consistent reporting of units, and the universal use of isotope-labeled internal standards. Careful accounting for biological and methodological confounders is therefore essential to ensure valid interpretation of TMAO as a metabolic biomarker.

Accordingly, plasma or serum TMAO is preferable when the objective is to characterize systemic exposure and to support cardiometabolic risk assessment or prevention, whereas urinary TMAO, ideally from a twenty-four-hour collection, or alternatively from spot urine normalized to creatinine offers greater sensitivity to recent dietary intake as well as to total excretion. The analytical gold standard remains isotope-dilution liquid chromatography, tandem mass spectrometry, and broader adoption of standardization guidelines would likely enhance reproducibility at the population level (29). The major biological and methodological confounders influencing circulating and urinary TMAO interpretation are summarized in **Table 1**.

**Table 1:** Key biological and methodological confounders influencing circulating and urinary TMAO interpretation (8, 35).

Confounder	Influence on TMAO levels	Relevance for interpretation
Dietary intake	Acute and habitual consumption of fish, seafood, red meat, eggs, and dairy directly affects circulating and urinary TMAO	May reflect recent exposure rather than chronic metabolic status
Gut microbiota composition	Determines the capacity for TMA production from dietary precursors	Contributes to inter-individual variability across age groups
Renal function	Reduced glomerular filtration rate leads to higher systemic TMAO concentrations	Major determinant of circulating TMAO; requires adjustment in analyses
Age	Pediatric vs adult populations show different metabolic and microbiota profiles	Influences interpretation across the life course
Gender	Hormonal and metabolic differences may modulate FMO3 activity and TMAO levels	Potential source of biological heterogeneity
Fasting status	Non-fasting samples may reflect postprandial TMAO fluctuations	Affects comparability between studies
Sample type	Plasma/serum vs urine reflect systemic exposure vs excretion	Determines the clinical and research utility of TMAO
Preanalytical handling	Storage temperature, processing delay, freeze–thaw cycles	Impacts measurement reliability

## 3 Mechanistic pathways underlying the cardiorenal-metabolic effects of TMAO

TMAO has been most extensively investigated in relation to cardiovascular diseases (42). Elevated circulating levels have been associated with atherosclerosis, myocardial infarction, stroke, and heart failure (43–45). Mechanistic evidence suggests that TMAO may contribute to impaired cholesterol metabolism, endothelial dysfunction, foam cell formation, and enhanced platelet reactivity, thereby promoting a pro-atherogenic and pro-thrombotic environment (46, 47). Prospective cohort studies and meta-analyses indicate that elevated TMAO levels predict recurrent cardiovascular events and mortality independently of traditional risk factors, and in populations with heart failure, they have been consistently linked to poorer prognosis (43). Although these associations are robust, causality remains under debate, and ongoing clinical trials targeting microbial TMA generation or hepatic FMO3 activity aim to clarify the therapeutic potential of this pathway (48).

In chronic kidney disease (CKD), the interplay between TMAO and renal function appears to be bidirectional (49). Impaired renal clearance contributes to systemic accumulation of TMAO, while experimental models indicate that elevated concentrations may, in turn, exacerbate renal injury by promoting interstitial fibrosis, oxidative stress, and low-grade inflammation (50–52). Observational data consistently reveal higher circulating TMAO levels in individuals with CKD, and quantitative syntheses report associations with accelerated decline in estimated glomerular filtration rate (eGFR) and increased all-cause mortality (53, 54). This dual nature: TMAO as both a surrogate marker of reduced excretory capacity and a potential pathogenic mediator, poses interpretative challenges (25). Furthermore, variability in dietary choline and carnitine intake, interindividual differences in gut microbiota composition, and methodological heterogeneity across studies limit comparability and causal inference (55). These complexities underscore the necessity of well-controlled prospective trials to determine whether therapeutic modulation of TMAO generation or clearance could mitigate renal functional decline (56, 57).

The relationship between TMAO and metabolic disorders has also gained considerable scientific attention (58). Individuals with type 2 diabetes (T2D) frequently exhibit higher circulating TMAO concentrations compared with healthy controls (59, 60) and several prospective cohorts have reported an increased risk of incident T2D associated with elevated baseline TMAO levels (61, 62). Proposed mechanisms include impaired insulin signaling, altered bile acid metabolism, enhanced hepatic gluconeogenesis, and chronic low-grade inflammation, all consistent with the metabolic dysfunction characteristic of obesity (63). However, findings across studies remain partly inconsistent, as some cohorts have reported null associations, suggesting that dietary patterns, genetic variability in FMO3 activity, and interindividual differences in gut microbiota composition may modulate these relationships. Overall, current evidence supports TMAO as a biomarker of metabolic dysregulation rather than a firmly established causal mediator (64).

Evidence linking TMAO to cancer remains limited and inconsistent. A large population-based study found no association between serum TMAO concentrations and overall cancer incidence, arguing against a generalized carcinogenic role (65). Nonetheless, site-specific associations have been reported, particularly for distal colorectal cancer, where higher levels of TMAO and its precursors have been correlated with increased risk (66). Preclinical data further suggest that TMAO may promote tumor cell proliferation and migration in prostate cancer through mechanisms involving oxidative stress and MAPK signaling pathways; however, these findings are preliminary and require validation in prospective human cohorts (67). Collectively, current evidence indicates that the relationship between TMAO and malignancy is likely context-dependent rather than universal (68).

Emerging research has also implicated TMAO in neurological and inflammatory disorders (69). Elevated circulating TMAO levels have been associated with a greater burden of white matter lesions, cognitive decline, and poorer functional outcomes following ischemic stroke (70). Experimental studies demonstrate that TMAO can trigger microglial activation and neuroinflammatory responses, providing a plausible mechanistic basis for these associations (71). More broadly, TMAO appears to modulate systemic inflammatory pathways, with reported links to hypertension, vascular stiffness, and circulating inflammatory biomarkers (72). These findings extend the potential pathophysiological relevance of TMAO beyond cardiometabolic disease; however, human evidence remains limited and often heterogeneous (3). Further longitudinal studies with standardized assessment protocols are warranted to elucidate the role of TMAO in neurological and inflammatory conditions (68).

## 4 Circulating TMAO in obesity

In pediatric cohorts, circulating and urinary TMAO have begun to be characterized, with several cross-sectional studies reporting higher serum TMAO concentrations in children with obesity compared to normal-weight controls, along with positive correlations with adiposity indices such as BMI, waist circumference, and waist-to-height ratio (73). A multicenter Romanian study further identified TMAO as an independent predictor of these anthropometric parameters and linked elevated levels to markers of subclinical vascular injury, suggesting that microbial methylamines represent measurable obesity-related exposures from early life (12). Urinary TMAO also reflects habitual dietary patterns in children, particularly the intake of fish, meat, and eggs and has been shown to decrease following lifestyle interventions in prepubertal children with obesity (73). However, it should be noted that most available pediatric data are derived from cross-sectional analyses with relatively small sample sizes and limited longitudinal follow-up, which constrains causal inference and the assessment of long-term metabolic trajectories. These findings support the concept that pediatric TMAO levels are influenced by both diet and gut microbiota composition and may be responsive to behavioral modification (12, 74).

Links between TMAO and inflammation, lipid metabolism, vascular stiffness, and hepatic alterations are increasingly reported in younger populations, although sample sizes remain smaller than those in adult studies (16). Elevated TMAO levels in children with obesity have been shown to co-vary with carotid intima–media thickness, pulse wave velocity, and central blood pressure, findings that parallel evidence of early arterial stiffening as a subclinical cardiovascular phenotype of risk in pediatric obesity (12). Some epidemiological datasets suggest that TMAO precursors (e.g., choline and betaine) may correlate more strongly than TMAO itself with adverse inflammatory and cardiometabolic profiles in children, highlighting nuances in biomarker interpretation and pathway specificity (14). Pediatric hepatic steatosis, currently referred to as MAFLD is highly prevalent in obesity; however, direct pediatric evidence linking TMAO to liver disease defined by biopsy or elastography remains scarce (75). Contemporary syntheses and consensus statements on MAFLD emphasize its high prevalence and metabolic determinants without establishing a specific causal role for TMAO (32).

In adults, the evidence base encompasses both cross-sectional and longitudinal investigations (58). Multiple cohort studies and meta-analyses have reported elevated TMAO concentrations in individuals with type 2 diabetes and metabolic syndrome, as well as prospective associations with incident diabetes and adverse cardiovascular outcomes (60, 76). Diet represents a major determinant of circulating TMAO levels: chronic consumption of red meat increases systemic TMAO through enhanced microbial and host production, whereas predominantly plant-based dietary patterns are generally associated with lower levels, underscoring exposure variability across studies (77). Overall, adult data support TMAO as an enriched biomarker of cardiometabolic risk, although causal inference remains limited pending interventional trials directly targeting microbial TMA generation or hepatic FMO3 activity (57).

The emerging evidence positions TMAO as an important metabolic intermediary that connects higher BMI with metabolic impairment and cardiovascular pathology (58). Elevated circulating TMAO concentrations show a robust relationship with central adiposity, supporting its role in the cascade of obesity-associated metabolic alterations. Its interaction with resistin points to a combined contribution to insulin resistance and cardiometabolic complications, underscoring their relevance in evaluating overall metabolic risk (76). Moreover, TMAO has been identified as an independent predictor of obesity, with a significant interaction effect that implies a more intricate, interdependent relationship rather than isolated action (12, 76). This interplay suggests a synergistic contribution to obesity susceptibility and highlights the value of jointly assessing these biomarkers for enhanced cardiometabolic risk prediction.

## 5 Circulating TMAO in type 2 diabetes

Over recent years, numerous studies have examined the relationship between TMAO and type 2 diabetes mellitus, proposing its involvement both as a marker of metabolic dysfunction and as a potential contributor to disease pathophysiology. Associations have been reported between circulating TMAO concentrations and reduced insulin sensitivity, reflected by higher HOMA-IR values, elevated HbA1c levels, increased waist circumference, and greater central adiposity (32). These observations support the relevance of TMAO as an indicator of adverse metabolic profiles in individuals with excess body weight and insulin resistance. However, existing evidence remains heterogeneous, and it is not yet clear whether elevated TMAO represents a causal mediator of glucose dysregulation or predominantly reflects upstream influences such as dietary intake, renal function, and gut microbiota composition. This distinction is critical for clinical interpretation, as it determines whether TMAO should be viewed primarily as a risk marker or as a therapeutic target in the prevention and management of type 2 diabetes (32).

Moreover, one of the most robust observational studies demonstrated that individuals with elevated serum TMAO levels had more than twice the risk of developing diabetes compared to those with lower TMAO concentrations, independent of confounding factors and FMO3 gene polymorphism (78). These findings were supported by the prospective Guangzhou Nutrition and Health Study, in which Li et al. followed middle-aged and older adults for nearly nine years and observed that individuals with higher serum TMAO levels had a significantly increased risk of developing type 2 diabetes, as well as a progressive rise in fasting blood glucose over time, even after adjustment for multiple confounding factors (8). These findings were supported by the prospective Guangzhou Nutrition and Health Study, in which Li et al. followed middle-aged and older adults for nearly nine years and observed that individuals with higher serum TMAO levels had a significantly increased risk of developing type 2 diabetes, as well as a progressive rise in fasting blood glucose over time, even after adjustment for multiple confounding factors (35). As evidence connecting TMAO with the onset of diabetes continues to accumulate, though not always consistently, interest has shifted toward determining whether TMAO can serve as a reliable biomarker for future diabetes risk (11). Longitudinal cohort investigations, which overcome many of the limitations of cross-sectional designs, have provided insight into the temporal link between circulating TMAO at baseline and the later emergence of diabetes. For type 2 diabetes specifically, two independent groups documented that individuals with elevated baseline TMAO exhibited a greater likelihood of developing the disease over time (8, 79). Huang et al. further demonstrated that not only initial TMAO concentrations but also their longitudinal trajectories predicted incident type 2 diabetes (79).

Conversely, even though TMAO remained associated with parameters of insulin resistance, including HOMA index and fasting insulin, other prospective analyses failed to detect such a relationship (35, 80). Adding another layer of complexity, Papandreou et al. observed an inverse association in which higher TMAO concentrations appeared to correlate with a reduced risk of developing diabetes in the future (34). This unexpected result underscores the intricate physiology of TMAO and raises the possibility that context-dependent or compensatory mechanisms may modulate its metabolic effects. Moreover, the pronounced intra- and inter-individual variability in TMAO levels documented among patients with diabetes may further contribute to the heterogeneity reported across studies (81). Friedrich et al. offered a potential explanation for these divergent findings, showing that the increased diabetes risk linked to higher TMAO occurred exclusively in women, with no parallel effect observed in men (82).

Evidence linking TMAO to diabetes dates back to 1998, when Messana et al. first reported that individuals with diabetes exhibited markedly higher urinary TMAO levels, a finding that persisted regardless of metabolic control, glucosuria, or HbA1c values (83). Multiple investigations expanded these observations, documenting significant associations between circulating TMAO concentrations and diabetes across several types of cohorts, including large population-based samples, as well as studies enrolling patients with established cardiovascular conditions (84, 85). Although observational research cannot definitively establish causality, consistently identifying biomarkers that track with disease across diverse populations remains fundamental for defining potential therapeutic targets and improving risk stratification.

## 6 Future perspectives

Although the adult literature has established a consistent link between TMAO, obesity, and type 2 diabetes, pediatric evidence remains fragmented and preliminary, with heterogeneous findings. Most studies conducted in children are cross-sectional, based on small sample sizes, and often lack methodological standardization in biospecimen collection and analysis (fasting status, adjustment for renal function, or recent dietary intake). The absence of longitudinal data limits our ability to determine whether TMAO acts as an early determinant of metabolic disturbances or merely reflects concurrent dietary and microbial exposures. Furthermore, age-specific factors, such as puberty, metabolic plasticity, and gut microbiome maturation, add layers of complexity and highlight the need for age-tailored research.

In this context, well-powered prospective, multicenter studies with repeated measures and comprehensive phenotyping (including vascular, inflammatory, hepatic, and body composition parameters) are essential to clarify the longitudinal dynamics of TMAO throughout childhood and adolescence. Such designs would allow for the evaluation of TMAO as a predictive biomarker for the future development of cardiometabolic pathologies, including insulin resistance, arterial stiffness, and early endothelial dysfunction. Incorporating TMAO into risk prediction models, alongside established metrics such as BMI, lipid profile, HOMA-IR, and inflammatory markers, could improve early risk stratification and guide preventive interventions well before overt disease onset.

Moreover, nutritional and microbiota-targeted interventions could test the causal hypothesis that reducing TMAO concentrations positively influences cardiometabolic health trajectories. In the long term, TMAO may evolve from a passive biomarker of metabolic exposure to an active predictive and therapeutic target, enabling more refined strategies for early prevention and personalized management of metabolic and cardiovascular disease. Integrating pediatric and adult data is therefore crucial to building a comprehensive, life-course perspective on TMAO biology and its potential role in shaping lifelong cardiometabolic risk.

## 7 Conclusions

Integrating evidence from both pediatric and adult populations adds substantial value to understanding the complex role of TMAO in the pathogenesis of obesity and type 2 diabetes mellitus. The pediatric–adult comparison highlights a clear life-course gradient, in which elevated TMAO levels in children and adolescents appear to reflect early, potentially modifiable metabolic alterations closely linked to dietary exposure and gut microbiota composition. In contrast, adult data consistently associate higher TMAO concentrations with visceral adiposity, chronic low-grade inflammation, impaired metabolic regulation, and established cardiometabolic complications. This age-dependent pattern underscores the importance of considering developmental stage when interpreting TMAO-related findings.

From a translational perspective, these observations support the concept that TMAO may function primarily as an early biomarker of metabolic vulnerability in pediatric populations, while in adults it may increasingly reflect cumulative metabolic burden and disease progression. Distinguishing between these roles is central to clinical interpretation and may inform the timing and targets of preventive strategies. Importantly, the life-course framework presented in this review suggests that early modulation of diet–microbiota interactions could influence long-term cardiometabolic trajectories.

Given the escalating global burden and progressively earlier onset of obesity and type 2 diabetes, there is a pressing need for biomarkers that capture both metabolic disturbance and responsiveness to intervention. Positioned at the intersection of diet, gut microbiota, hepatic metabolism, and renal clearance, TMAO represents a promising candidate for risk stratification and personalized prevention strategies. However, its transition from an associative marker to a clinically actionable tool will require well-designed longitudinal and interventional studies, particularly those addressing causality, key confounders, and age-specific effects. Such evidence will be essential to define the future clinical relevance of TMAO in the prevention and management of metabolic disease across the lifespan.

## References

[1] Liu Y Dai M . Trimethylamine N-oxide generated by the gut microbiota is associated with vascular inflammation: new insights into atherosclerosis. Mediators Inflamm. (2020) 2020:4634172. doi: 10.1155/2020/4634172, PMID: 32148438 PMC7048942

[2] Caradonna E Abate F Schiano E Paparella F Ferrara F Vanoli E . Trimethylamine-N-oxide (TMAO) as a rising-star metabolite: implications for human health. Metabolites. (2025) 15:220. doi: 10.3390/metabo15040220, PMID: 40278349 PMC12029716

[3] Koeth RA Wang Z Levison BS Buffa JA Org E Sheehy BT . Intestinal microbiota metabolism of L-carnitine, a nutrient in red meat, promotes atherosclerosis. Nat Med. (2013) 19:576–85. doi: 10.1038/nm.3145, PMID: 23563705 PMC3650111

[4] Rajakovich LJ Fu B Bollenbach M Balskus EP . Elucidation of an anaerobic pathway for metabolism of l-carnitine-derived γ-butyrobetaine to trimethylamine in human gut bacteria. Proc Natl Acad Sci U S A. (2021) 118:e2101498118. doi: 10.1073/pnas.2101498118, PMID: 34362844 PMC8364193

[5] Zhu W Buffa JA Wang Z Warrier M Schugar R Shih DM . Flavin monooxygenase 3, the host hepatic enzyme in the metaorganismal trimethylamine N-oxide-generating pathway, modulates platelet responsiveness and thrombosis risk. J Thromb Haemost. (2018) 16:1857–72. doi: 10.1111/jth.14234, PMID: 29981269 PMC6156942

[6] Zhang Y Wang Y Ke B Du J . TMAO: how gut microbiota contributes to heart failure. Transl Res. (2021) 228:109–25. doi: 10.1016/j.trsl.2020.08.007, PMID: 32841736

[7] Li X Hong J Wang Y Pei M Wang L Gong Z . Trimethylamine-N-oxide pathway: A potential target for the treatment of MAFLD. Front Mol Biosci. (2021) 8:733507. doi: 10.3389/fmolb.2021.733507, PMID: 34660695 PMC8517136

[8] Li SY Chen S Lu XT Fang AP Chen YM Huang RZ . Serum trimethylamine-N-oxide is associated with incident type 2 diabetes in middle-aged and older adults: a prospective cohort study. J Transl Med. (2022) 20:374. doi: 10.1186/s12967-022-03581-7, PMID: 35982495 PMC9389664

[9] Kalagi NA Thota RN Stojanovski E Alburikan KA Garg ML . Association between plasma trimethylamine N-oxide levels and type 2 diabetes: A case control study. Nutrients. (2022) 14:2093. doi: 10.3390/nu14102093, PMID: 35631234 PMC9148165

[10] Kong L Zhao Q Jiang X Hu J Jiang Q Sheng L . Trimethylamine N-oxide impairs β-cell function and glucose tolerance. Nat Commun. (2024) 15:2526. doi: 10.1038/s41467-024-46829-0, PMID: 38514666 PMC10957989

[11] Jaworska K Kuś M Ufnal M . TMAO and diabetes: from the gut feeling to the heart of the problem. Nutr Diabetes. (2025) 15:21. doi: 10.1038/s41387-025-00377-8, PMID: 40393987 PMC12092638

[12] Mihuta MS Paul C Borlea A Roi CM Pescari D Velea-Barta OA . Connections between serum Trimethylamine N-Oxide (TMAO), a gut-derived metabolite, and vascular biomarkers evaluating arterial stiffness and subclinical atherosclerosis in children with obesity. Front Endocrinol (Lausanne). (2023) 14:1253584. doi: 10.3389/fendo.2023.1253584, PMID: 37850094 PMC10577381

[13] Huang PY Hsu BG Lai YH Wang CH Tsai JP . Serum trimethylamine *N*-oxide level is positively associated with aortic stiffness measured by carotid-femoral pulse wave velocity in patients undergoing maintenance hemodialysis. Toxins (Basel). (2023) 15:572. doi: 10.3390/toxins15090572, PMID: 37755998 PMC10538077

[14] Andraos S Jones B Lange K Clifford SA Thorstensen EB Kerr JA . Trimethylamine N-oxide (TMAO) is not associated with cardiometabolic phenotypes and inflammatory markers in children and adults. Curr Dev Nutr. (2020) 5:nzaa179. doi: 10.1093/cdn/nzaa179, PMID: 33501405 PMC7813154

[15] World Health Organization . Obesity and overweight. Fact sheet. Geneva: World Health Organization (2021).

[16] Iorra FQ Rodrigues PG Bock PM Guahnon MP Eller S de Oliveira TF . Gut microbiota metabolite TMAO and adolescent cardiometabolic health: A cross-sectional analysis. J Endocr Soc. (2025) 9:bvaf055. doi: 10.1210/jendso/bvaf055, PMID: 40242209 PMC12000724

[17] Wang Z Klipfell E Bennett BJ Koeth R Levison BS Dugar B . Gut flora metabolism of phosphatidylcholine promotes cardiovascular disease. Nature. (2011) 472:57–63. doi: 10.1038/nature09922, PMID: 21475195 PMC3086762

[18] Van Parys A Karlsson T Vinknes KJ Olsen T Øyen J Dierkes J . Food sources contributing to intake of choline and individual choline forms in a norwegian cohort of patients with stable angina pectoris. Front Nutr. (2021) 8:676026. doi: 10.3389/fnut.2021.676026, PMID: 34055860 PMC8160433

[19] Kalnins G Kuka J Grinberga S Makrecka-Kuka M Liepinsh E Dambrova M . Structure and function of cutC choline lyase from human microbiota bacterium klebsiella pneumoniae. J Biol Chem. (2015) 290:21732–40. doi: 10.1074/jbc.M115.670471, PMID: 26187464 PMC4571895

[20] Quareshy M Shanmugam M Townsend E Jameson E Bugg TDH Cameron AD . Structural basis of carnitine monooxygenase CntA substrate specificity, inhibition, and intersubunit electron transfer. J Biol Chem. (2021) 296:100038. doi: 10.1074/jbc.RA120.016019, PMID: 33158989 PMC7948474

[21] Craciun S Balskus EP . Microbial conversion of choline to trimethylamine requires a glycyl radical enzyme. Proc Natl Acad Sci U S A. (2012) 109:21307–12. doi: 10.1073/pnas.1215689109, PMID: 23151509 PMC3535645

[22] Yeung CK Adman ET Rettie AE . Functional characterization of genetic variants of human FMO3 associated with trimethylaminuria. Arch Biochem Biophys. (2007) 464:251–9. doi: 10.1016/j.abb.2007.04.014, PMID: 17531949 PMC2039921

[23] Fennema D Phillips IR Shephard EA . Trimethylamine and trimethylamine N-oxide, a flavin-containing monooxygenase 3 (FMO3)-mediated host-microbiome metabolic axis implicated in health and disease. Drug Metab Dispos. (2016) 44:1839–50. Erratum in: Drug Metab Dispos. 2016 Dec;44(12):1949. doi: 10.1124/dmd.116.070615, PMID: 27190056 PMC5074467

[24] Miao J Ling AV Manthena PV Gearing ME Graham MJ Crooke RM . Flavin-containing monooxygenase 3 as a potential player in diabetes-associated atherosclerosis. Nat Commun. (2015) 6:6498. doi: 10.1038/ncomms7498, PMID: 25849138 PMC4391288

[25] Cho CE Taesuwan S Malysheva OV Bender E Tulchinsky NF Yan J . Trimethylamine-N-oxide (TMAO) response to animal source foods varies among healthy young men and is influenced by their gut microbiota composition: A randomized controlled trial. Mol Nutr Food Res. (2017) 61. doi: 10.1002/mnfr.201600324, PMID: 27377678

[26] Wang Z Tang WHW O’Connell T Garcia E Jeyarajah EJ Li XS . Circulating trimethylamine N-oxide levels following fish or seafood consumption. Eur J Nutr. (2022) 61:2357–64. doi: 10.1007/s00394-022-02803-4, PMID: 35113194 PMC9283263

[27] Bergman P . Serum trimethylamine-N-oxide is strongly related to renal function and predicts outcome in chronic kidney disease. PloS One. (2016). doi: 10.1371/journal.pone.0141738, PMID: 26751065 PMC4709190

[28] Wang Z Levison BS Hazen JE Donahue L Li XM Hazen SL . Measurement of trimethylamine-N-oxide by stable isotope dilution liquid chromatography tandem mass spectrometry. Anal Biochem. (2014) 455:35–40. doi: 10.1016/j.ab.2014.03.016, PMID: 24704102 PMC4167037

[29] Mafune A Iwamoto T Tsutsumi Y Nakashima A Yamamoto I Yokoyama K . Associations among serum trimethylamine-N-oxide (TMAO) levels, kidney function and infarcted coronary artery number in patients undergoing cardiovascular surgery: a cross-sectional study. Clin Exp Nephrol. (2016) 20:731–9. doi: 10.1007/s10157-015-1207-y, PMID: 26676906 PMC5050242

[30] Hefni ME Bergström M Lennqvist T Fagerström C Witthöft CM . Simultaneous quantification of trimethylamine N-oxide, trimethylamine, choline, betaine, creatinine, and propionyl-, acetyl-, and L-carnitine in clinical and food samples using HILIC-LC-MS. Anal Bioanal Chem. (2021) 413:5349–60. doi: 10.1007/s00216-021-03509-y, PMID: 34258650 PMC8405501

[31] Erickson ML Malin SK Wang Z Brown JM Hazen SL Kirwan JP . Effects of lifestyle intervention on plasma trimethylamine N-oxide in obese adults. Nutrients. (2019) 11:179. doi: 10.3390/nu11010179, PMID: 30654453 PMC6356515

[32] Pescari D Mihuta MS Bena A Stoian D . Independent predictors of circulating trimethylamine N-oxide (TMAO) and resistin levels in subjects with obesity: associations with carotid intima-media thickness and metabolic parameters. Nutrients. (2025) 17:798. doi: 10.3390/nu17050798, PMID: 40077669 PMC11902032

[33] Andrikopoulos P Aron-Wisnewsky J Chakaroun R Myridakis A Forslund SK Nielsen T . Evidence of a causal and modifiable relationship between kidney function and circulating trimethylamine *N*-oxide. Nat Commun. (2023) 14:5843. doi: 10.1038/s41467-023-39824-4, PMID: 37730687 PMC10511707

[34] Papandreou C Bulló M Zheng Y Ruiz-Canela M Yu E Guasch-Ferré M . Plasma trimethylamine-N-oxide and related metabolites are associated with type 2 diabetes risk in the Prevención con Dieta Mediterránea (PREDIMED) trial. Am J Clin Nutr. (2018) 108:163–73. doi: 10.1093/ajcn/nqy058. ISSN 0002-9165., PMID: 29982310 PMC6862602

[35] Lemaitre RN Jensen PN Wang Z Fretts AM McKnight B Nemet I . Association of trimethylamine N-oxide and related metabolites in plasma and incident type 2 diabetes: the cardiovascular health study. JAMA Netw Open. (2021) 4:e2122844. doi: 10.1001/jamanetworkopen.2021.22844, PMID: 34448864 PMC8397925

[36] Pelletier CC Croyal M Ene L Aguesse A Billon-Crossouard S Krempf M . Elevation of trimethylamine-N-oxide in chronic kidney disease: contribution of decreased glomerular filtration rate. Toxins (Basel). (2019) 11:635. doi: 10.3390/toxins11110635, PMID: 31683880 PMC6891811

[37] Yin X Gibbons H Rundle M Frost G McNulty BA Nugent AP . The relationship between fish intake and urinary trimethylamine-N-oxide. Mol Nutr Food Res. (2020) 64:e1900799. doi: 10.1002/mnfr.201900799, PMID: 31863680

[38] Tang KW Toh QC Teo BW . Normalisation of urinary biomarkers to creatinine for clinical practice and research--when and why. Singapore Med J. (2015) 56:7–10. doi: 10.11622/smedj.2015003, PMID: 25640093 PMC4325562

[39] Leslie SW Rathi BM Sharif S . 24-hour urine collection and analysis. In: StatPearls. StatPearls Publishing, Treasure Island (FL (2025). Available online at: https://www.ncbi.nlm.nih.gov/books/NBK482482/ (Accessed October 06, 2024). 29494089

[40] Abraham K Penczynski K Monien BH Bergau N Knüppel S Weikert C . Risks of misinterpretation of biomarker measurements in spot urine adjusted for creatinine - A problem especially for studies comparing plant based with omnivorous diets. Int J Hyg Environ Health. (2023) 249:114142. doi: 10.1016/j.ijheh.2023.114142, PMID: 36842230

[41] KoniecznyRA KuliczkowskiW . Trimethylamine N-oxide in cardiovascular disease. Adv Clin Exp Med. (2022) 31:913–25. doi: 10.17219/acem/147666, PMID: 35438848

[42] Guasti L Galliazzo S Molaro M Visconti E Pennella B Gaudio GV . TMAO as a biomarker of cardiovascular events: a systematic review and meta-analysis. Intern Emerg Med. (2021) 16:201–7. doi: 10.1007/s11739-020-02470-5, PMID: 32779113

[43] Yamashita T Yoshida N Emoto T Hirata KI . Unraveling the Effects of Trimethylamine N-Oxide on Stroke: “The lower, the better? J Atheroscler Thromb. (2021) 28:314–6. doi: 10.5551/jat.ED142, PMID: 32999209 PMC8147014

[44] Jing L Zhang H Xiang Q Hu H Zhai C Xu S . Role of trimethylamine N-oxide in heart failure. Rev Cardiovasc Med. (2024) 25:240. doi: 10.31083/j.rcm2507240, PMID: 39139438 PMC11317343

[45] Zhu W Gregory JC Org E Buffa JA Gupta N Wang Z . Gut microbial metabolite TMAO enhances platelet hyperreactivity and thrombosis risk. Cell. (2016) 165:111–24. doi: 10.1016/j.cell.2016.02.011, PMID: 26972052 PMC4862743

[46] Berger M Kleber ME Delgado GE März W Andreas M Hellstern P . Trimethylamine N-oxide and adenosine diphosphate-induced platelet reactivity are independent risk factors for cardiovascular and all-cause mortality. Circ Res. (2020) 126:660–2. doi: 10.1161/CIRCRESAHA.119.316214, PMID: 31958034

[47] Jaworska K Kopacz W Koper M Ufnal M . Microbiome-derived trimethylamine N-oxide (TMAO) as a multifaceted biomarker in cardiovascular disease: challenges and opportunities. Int J Mol Sci. (2024) 25:12511. doi: 10.3390/ijms252312511, PMID: 39684223 PMC11641139

[48] Tang WH Hazen SL . The contributory role of gut microbiota in cardiovascular disease. J Clin Invest. (2014) 124:4204–11. doi: 10.1172/JCI72331, PMID: 25271725 PMC4215189

[49] Su JQ Wu XQ Wang Q Xie BY Xiao CY Su HY . The microbial metabolite trimethylamine N-oxide and the kidney diseases. Front Cell Infect Microbiol. (2025) 15:1488264. doi: 10.3389/fcimb.2025.1488264, PMID: 40134790 PMC11933022

[50] Kapetanaki S Kumawat AK Persson K Demirel I . The fibrotic effects of TMAO on human renal fibroblasts is mediated by NLRP3, caspase-1 and the PERK/akt/mTOR pathway. Int J Mol Sci. (2021) 22:11864. doi: 10.3390/ijms222111864, PMID: 34769294 PMC8584593

[51] Zhang W Miikeda A Zuckerman J Jia X Charugundla S Zhou Z . Inhibition of microbiota-dependent TMAO production attenuates chronic kidney disease in mice. Sci Rep. (2021) 11:518. doi: 10.1038/s41598-020-80063-0, PMID: 33436815 PMC7804188

[52] Stubbs JR House JA Ocque AJ Zhang S Johnson C Kimber C . Serum trimethylamine-N-oxide is elevated in CKD and correlates with coronary atherosclerosis burden. J Am Soc Nephrol. (2016) 27:305–13. doi: 10.1681/ASN.2014111063, PMID: 26229137 PMC4696571

[53] Li XS Obeid S Klingenberg R Gencer B Mach F Räber L . Gut microbiota-dependent trimethylamine N-oxide in acute coronary syndromes: a prognostic marker for incident cardiovascular events beyond traditional risk factors. Eur Heart J. (2017) 38:814–24. doi: 10.1093/eurheartj/ehw582, PMID: 28077467 PMC5837488

[54] Ahmadmehrabi S Tang WHW . Gut microbiome and its role in cardiovascular diseases. Curr Opin Cardiol. (2017) 32:761–6. doi: 10.1097/HCO.0000000000000445, PMID: 29023288 PMC5746314

[55] Organ CL Otsuka H Bhushan S Wang Z Bradley J Trivedi R . Choline diet and its gut microbe-derived metabolite, trimethylamine N-oxide, exacerbate pressure overload-induced heart failure. Circ Heart Fail. (2016) 9:e002314. doi: 10.1161/CIRCHEARTFAILURE.115.002314, PMID: 26699388 PMC4943035

[56] Antza C Stabouli S Kotsis V . Gut microbiota in kidney disease and hypertension. Pharmacol Res. (2018) 130:198–203. doi: 10.1016/j.phrs.2018.02.028, PMID: 29496593

[57] Janeiro MH Ramírez MJ Milagro FI Martínez JA Solas M . Implication of trimethylamine N-oxide (TMAO) in disease: potential biomarker or new therapeutic target. Nutrients. (2018) 10:1398. doi: 10.3390/nu10101398, PMID: 30275434 PMC6213249

[58] Dambrova M Latkovskis G Kuka J Strele I Konrade I Grinberga S . Diabetes is associated with higher trimethylamine N-oxide plasma levels. Exp Clin Endocrinol Diabetes. (2016) 124:251–6. doi: 10.1055/s-0035-1569330, PMID: 27123785

[59] Zhuang R Ge X Han L Yu P Gong X Meng Q . Gut microbe-generated metabolite trimethylamine N-oxide and the risk of diabetes: A systematic review and dose-response meta-analysis. Obes Rev. (2019) 20:883–94. doi: 10.1111/obr.12843, PMID: 30868721

[60] Qi J You T Li J Pan T Xiang L Han Y . Circulating trimethylamine N-oxide and the risk of cardiovascular diseases: a systematic review and meta-analysis of 11 prospective cohort studies. J Cell Mol Med. (2018) 22:185–94. doi: 10.1111/jcmm.13307, PMID: 28782886 PMC5742728

[61] Chen S Henderson A Petriello MC Romano KA Gearing M Miao J . Trimethylamine N-oxide binds and activates PERK to promote metabolic dysfunction. Cell Metab. (2019) 30:1141–51. doi: 10.1016/j.cmet.2019.08.021, PMID: 31543404

[62] Schugar RC Shih DM Warrier M Helsley RN Burrows A Ferguson D . The TMAO-producing enzyme flavin-containing monooxygenase 3 regulates obesity and the beiging of white adipose tissue. Cell Rep. (2017) 19:2451–61. doi: 10.1016/j.celrep.2017.05.077. Erratum in: Cell Rep. 2017 Jul 5;20(1):279. doi: 10.1016/j.celrep.2017.06.053., PMID: 28636934 PMC5672822

[63] Cho CE Caudill MA . Trimethylamine-N-oxide: friend, foe, or simply caught in the cross-fire? Trends Endocrinol Metab. (2017) 28:121–30. doi: 10.1016/j.tem.2016.10.005, PMID: 27825547

[64] Rohrmann S Linseisen J Allenspach M von Eckardstein A Müller D . Plasma concentrations of trimethylamine-N-oxide are directly associated with dairy food consumption and low-grade inflammation in a german adult population. J Nutr. (2016) 146:283–9. doi: 10.3945/jn.115.220103, PMID: 26674761

[65] Bae S Ulrich CM Neuhouser ML Malysheva O Bailey LB Xiao L . Plasma choline metabolites and colorectal cancer risk in the Women’s Health Initiative Observational Study. Cancer Res. (2014) 74:7442–52. doi: 10.1158/0008-5472.CAN-14-1835, PMID: 25336191 PMC4268282

[66] Zhou Y Lv J Jin S Fu C Liu B Shen Y . Gut microbiota derived metabolite trimethylamine N-oxide influences prostate cancer progression via the p38/HMOX1 pathway. Front Pharmacol. (2025) 15:1526051. doi: 10.3389/fphar.2024.1526051, PMID: 39850572 PMC11754881

[67] Li T Chen Y Gua C Li X . Elevated circulating trimethylamine N-oxide levels contribute to endothelial dysfunction in aged rats through vascular inflammation and oxidative stress. Front Physiol. (2017) 8:350. doi: 10.3389/fphys.2017.00350, PMID: 28611682 PMC5447752

[68] Li D Ke Y Zhan R Liu C Zhao M Zeng A . Trimethylamine-N-oxide promotes brain aging and cognitive impairment in mice. Aging Cell. (2018) 17:e12768. doi: 10.1111/acel.12768, PMID: 29749694 PMC6052480

[69] Ren Z Mo L . Association between levels of trimethylamine N-oxide and cognitive dysfunction: a systematic review and meta-analysis. PeerJ. (2025) :13:e20000. doi: 10.7717/peerj.20000, PMID: 40936751 PMC12422268

[70] Han JH Rey FE Denu JM . Gut microbiota-derived metabolite trimethylamine N-oxide alters the host epigenome through inhibition of S-adenosylhomocysteine hydrolase. J Biol Chem. (2025) 301:110521. doi: 10.1016/j.jbc.2025.110521, PMID: 40716746 PMC12390942

[71] Wang M Li XS Wang Z de Oliveira Otto MC Lemaitre RN Fretts A . Trimethylamine N-oxide is associated with long-term mortality risk: the multi-ethnic study of atherosclerosis. Eur Heart J. (2023) 44:1608–18. doi: 10.1093/eurheartj/ehad089, PMID: 36883587 PMC10411925

[72] Leal-Witt MJ Llobet M Samino S Castellano P Cuadras D Jimenez-Chillaron JC . Lifestyle intervention decreases urine trimethylamine N-oxide levels in prepubertal children with obesity. Obes (Silver Spring). (2018) 26:1603–10. doi: 10.1002/oby.22271, PMID: 30204940

[73] Maros ME Cho CG Junge AG Kämpgen B Saase V Siegel F . Comparative analysis of machine learning algorithms for computer-assisted reporting based on fully automated cross-lingual RadLex mappings. Sci Rep. (2021) 11:5529. doi: 10.1038/s41598-021-85016-9, PMID: 33750857 PMC7970897

[74] Lin YC Liao FM Chao HC Chen AC Jeng YM Lin CC . Consensus statement on metabolic dysfunction-associated steatotic liver disease in children and adolescents from the joint TASL-TSPGHAN expert committee. JGH Open. (2025) 9:e70137. doi: 10.1002/jgh3.70137, PMID: 40520848 PMC12162361

[75] Schwimmer JB Biddinger SB Ibrahim SH . MASLD in children: integrating epidemiological trends with mechanistic and translational advances. J Clin Invest. (2025) 135:e186422. doi: 10.1172/JCI186422, PMID: 40590222 PMC12208537

[76] Wang Z Bergeron N Levison BS Li XS Chiu S Jia X . Impact of chronic dietary red meat, white meat, or non-meat protein on trimethylamine N-oxide metabolism and renal excretion in healthy men and women. Eur Heart J. (2019) 40:583–94. doi: 10.1093/eurheartj/ehy799, PMID: 30535398 PMC6374688

[77] Chen S Chen XY Huang ZH Fang AP Li SY Huang RZ . Correlation between serum trimethylamine-N-oxide and body fat distribution in middle-aged and older adults: a prospective cohort study. Nutr J. (2024) 23:70. doi: 10.1186/s12937-024-00974-w, PMID: 38982486 PMC11234726

[78] Shan Z Sun T Huang H Chen S Chen L Luo C . Association between microbiota-dependent metabolite trimethylamine-*N*-oxide and type 2 diabetes. Am J Clin Nutr. (2017) 106:888–94. doi: 10.3945/ajcn.117.157107, PMID: 28724646

[79] Huang Y Wu Y Zhang Y Bai H Peng R Ruan W . Dynamic changes in gut microbiota-derived metabolite trimethylamine-N-oxide and risk of type 2 diabetes mellitus: potential for dietary changes in diabetes prevention. Nutrients. (2024) 16:1711. doi: 10.3390/nu16111711, PMID: 38892643 PMC11174887

[80] Svingen GF Schartum-Hansen H Pedersen ER Ueland PM Tell GS Mellgren G . Prospective associations of systemic and urinary choline metabolites with incident type 2 diabetes. Clin Chem. (2016) 62:755–65. doi: 10.1373/clinchem.2015.250761, PMID: 26980210

[81] McEntyre CJ Lever M Chambers ST George PM Slow S Elmslie JL . Variation of betaine, N, N-dimethylglycine, choline, glycerophosphorylcholine, taurine and trimethylamine-N-oxide in the plasma and urine of overweight people with type 2 diabetes over a two-year period. Ann Clin Biochem. (2015) 52:352–60. doi: 10.1177/0004563214545346, PMID: 25013088

[82] Friedrich N Budde K Suhre K Völker U John U Felix SB . Sex differences in urine metabolites related with risk of diabetes using NMR spectroscopy: results of the study of health in pomerania. Metabolomics. (2015) 11:1405–15. doi: 10.1007/s11306-015-0795-6

[83] Messana I Forni F Ferrari F Rossi C Giardina B Zuppi C . Proton nuclear magnetic resonance spectral profiles of urine in type II diabetic patients. Clin Chem. (1998) 44:1529–34. doi: 10.1093/clinchem/44.7.1529, PMID: 9665433

[84] Gruppen EG Garcia E Connelly MA Jeyarajah EJ Otvos JD Bakker SJL . TMAO is associated with mortality: impact of modestly impaired renal function. Sci Rep. (2017) 7:13781. doi: 10.1038/s41598-017-13739-9, PMID: 29061990 PMC5653802

[85] Sanchez-Alcoholado L Castellano-Castillo D Jordán-Martínez L Moreno-Indias I Cardila-Cruz P Elena D . Role of gut microbiota on cardio-metabolic parameters and immunity in coronary artery disease patients with and without type-2 diabetes mellitus. Front Microbiol. (2017) 8:1936. doi: 10.3389/fmicb.2017.01936, PMID: 29051757 PMC5633746

